# *In situ* semi-quantitative imaging of intracellular metabolic interaction by confocal Raman microscopy

**DOI:** 10.1016/j.isci.2025.113558

**Published:** 2025-09-12

**Authors:** Wanying He, Minxiao Wang, Zhaoshan Zhong, Hao Chen, Shichuan Xi, Huan Zhang, Mengna Li, Wenhao Sun, Yan Zhang, Yun Wang, Xiaoxiao Guo, Lianfu Li, Zengfeng Du, Zhendong Luan, Chaolun Li, Xin Zhang

**Affiliations:** 1 Laoshan Laboratory, Qingdao 266237, China; 2 Key Laboratory of Ocean Observation and Forecasting, CAS Key Laboratory of Marine Geology and Environment & Center of Deep Sea Research, Institute of Oceanology, Center for Ocean Mega-Science, Chinese Academy of Sciences, Qingdao 266071, China; 3 University of Chinese Academy of Sciences, Beijing 100049, China; 4 CAS Key Laboratory of Marine Ecology and Environmental Sciences & Center of Deep Sea Research, Institute of Oceanology, Chinese Academy of Sciences, Qingdao 266071, China; 5 Laboratory for Marine Ecology and Environmental Science, Qingdao National Laboratory for Marine Science and Technology, Qingdao 266061, China; 6 National Laboratory of Biomacromolecules, CAS Center for Excellence in Biomacromolecules, Institute of Biophysics, Chinese Academy of Sciences, Beijing 100101, China; 7 Oxford Suzhou Centre for Advanced Research, University of OSCAR, Suzhou 215123, China; 8 South China Sea Institute of Oceanology, Chinese Academy of Sciences, Guangzhou 510301, China

**Keywords:** Biochemistry, Metabolomics

## Abstract

Non-destructive subcellular metabolite quantification can reveal critical insights into biological interactions (e.g., endosymbiont-host crosstalk). Therefore, we developed a multivariate semi-quantitative imaging method using internal standardization to resolve simultaneous subcellular distributions of multiple metabolites, leveraging confocal Raman microscopy’s (CRM’s) high spatial resolution. The method was applied to the endosymbiotic mussel *Gigantidas platifrons*, whose symbiotic interaction mechanism has not been elucidated because symbionts cannot be cultivated. The results showed that the aggregated distribution of distinct phenotypes of symbiont strains was characterized by different glycogen abundances, indicating niche-driven metabolic strategies. Our data may provide direct evidence suggesting that symbionts supply intermediates to the host for cholesterol synthesis, potentially via vesicular trafficking. This work demonstrates CRM’s capacity for comparative, spatially resolved metabolite quantification across cellular compartments. While semi-quantitative, CRM emerges as a powerful non-invasive tool for probing metabolic network dynamics and compartmentalization in challenging biological systems where traditional methods are limited.

## Highlights

• Multivariate semi-quantitative intracellular imaging by Raman microscopy
• Resolved subcellular distribution and content of multiple metabolites
• Reveals niche-driven metabolic strategies in endosymbiont
• The symbiont may produce cholesterol intermediates to the host

## Graphical abstract

**Figure:**
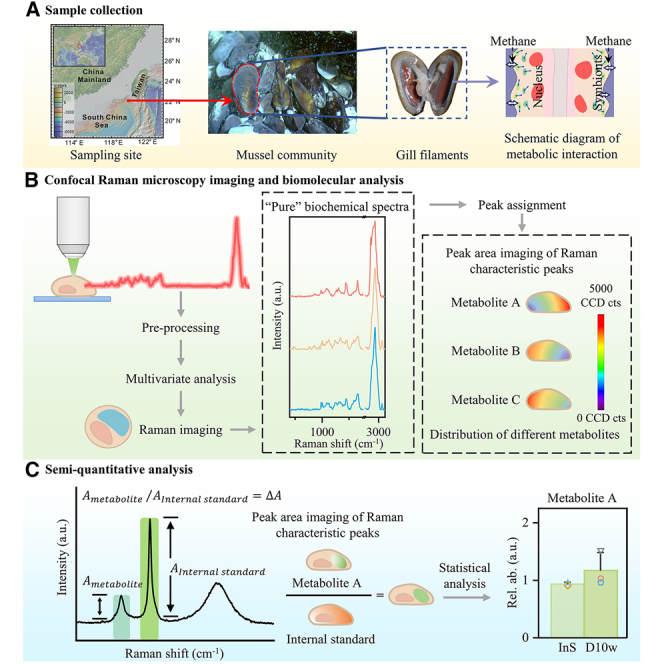

## Introduction

The symbiosis between bacteria and host cells is common in nature.^1^^,^^2^^,^^3^^,^^4^ Their counterparts establish strong interactions by sharing metabolites or cooperating to complete some metabolic pathways.^5^^,^^6^^,^^7^ Furthermore, some metabolites may serve as important signaling molecules that regulate crosstalk between cells. Thus, it is necessary to locate and quantify metabolites at the cellular scale to understand how energy and matter are transferred among organisms and how they are regulated,^4^^,^^5^ which is critical to the understanding of the host-microbe and microbe-microbe interactions.^4^^,^^8^^,^^9^ *Gigantidas platifrons* is a dominant species in hydrothermal vents and cold seeps. There are hundreds of methanotrophic bacteria dwelling inside the epithelial cells of the gill, referred to as bacteriocytes.^2^^,^^10^ Endosymbionts synthesize organic carbon through methane oxidation and formaldehyde assimilation; they may cooperate to complete the synthesis of sterols.^11^ Furthermore, cooperation between bacteriocytes and endosymbionts drives the formation and development of symbiotic cells and organs.^11^^,^^12^ Thus, *G. platifrons* could serve as a good model for testing metabolic interactions.

However, simultaneous qualitative and semi-quantitative estimation of metabolites at the subcellular scale remains a technical challenge. While mass spectrometry imaging has revolutionized molecular mapping through its label-free operation and multiplex detection capability, current implementations remain constrained by fundamental resolution thresholds (typically at micron scale) that limit subcellular metabolite tracking.^13^^,^^14^ Notably, recent studies have successfully achieved 600 nm pixel resolution in brain tissue studies through technological innovation,^15^ but the destruction of samples may further limit the utilization of rare samples, such as deep-sea lives.

Raman spectroscopy, a technique that uses inelastic light scattering to reflect chemical bond vibrations, provides a simple approach to nondestructively resolve complex biomolecular features at subcellular spatial scales with a lateral optical resolution of ∼350 nm.^15^ Raman spectroscopy has been widely used in the imaging of biological macromolecules,^16^^,^^17^ such as nucleic acids, proteins, and lipids in tissues or cells based on their characteristic peaks.^18^ However, the application of toolkits to recover the subcellular composition of metabolites in non-model cells remains challenging owing to the overlapping Raman peaks of multiple metabolites. Furthermore, most traditional Raman spectroscopy pipelines are designed for metabolite qualification, which makes comparative analysis of samples difficult. Currently, most Raman-based relative quantitative methods require isotopic labeling,^19^^,^^20^ which necessitates prior knowledge of the intracellular metabolic mechanisms and is difficult to implement for high-throughput analysis.^21^ Multivariate semi-quantitative analysis (MQA) is an efficient method to resolve complex metabolite mixtures.^22^^,^^23^ By combining the excellent resolving power of confocal Raman microscopy (CRM) and MQA, it is possible to understand the cellular details of metabolites.

To address the challenge of visualizing and quantifying multiple metabolites in a cell for non-model organisms, we developed an MQA method to analyze CRM imaging data with nanoscale resolution. A normalization library was established to quantify the multiple metabolites. The method was applied to the deep-sea mussel *G. platifrons* to verify the resolving power of the protocol for monitoring subcellular metabolic processes. We obtained *in situ* fixed samples and de-symbiosis samples by methane deprivation and compared the metabolic changes in the bacteriocytes to provide direct evidence of the interactions, including the contribution of farming or milking in deep-sea symbiosis.^11^^,^^12^^,^^24^^,^^25^

## Results and discussion

### Development of CRM for *G. platifrons*

**Figure 1:**
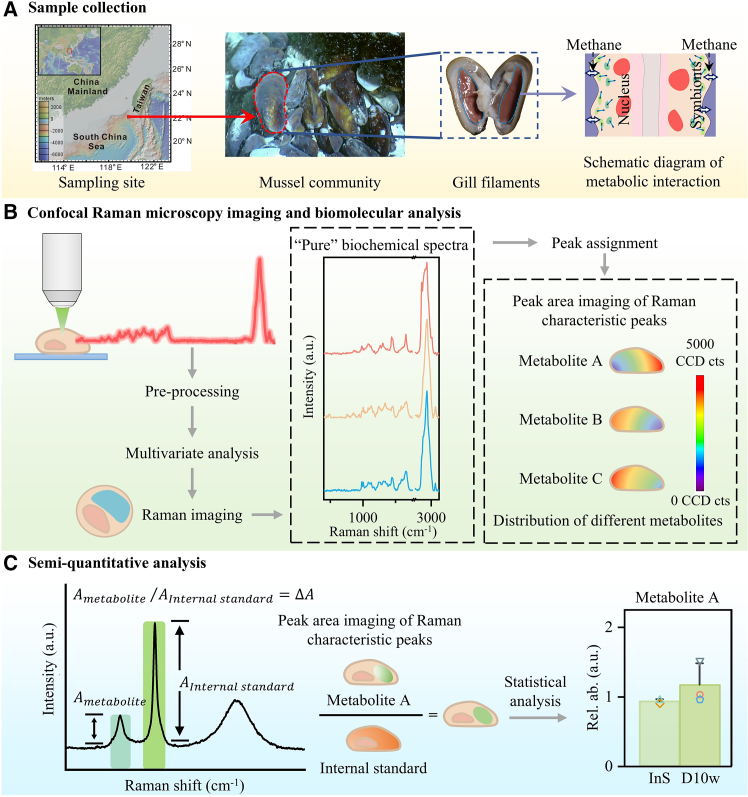
Overview of CRM imaging for MQA in intracellular metabolic interactions (A) Sample collection. The deep-sea mussels *G. platifrons* were sampled from the mussel communities in cold springs in the South China Sea. The gills of the mussels contained a large number of symbionts. (B) CRM imaging and biomolecular analysis. The spectral dataset of the entire gill cell was acquired for preprocessing and then subjected to multivariate analysis to elucidate its cellular composition. Subsequent peak assignment and metabolite characteristic peak imaging based on peak area was performed to obtain metabolite distribution information within the cell. (C) Semi-quantitative analysis of each component by calculating the normalized peak area (peak area ratio) of the Raman spectrum,^30^ that is, *A*_*metabolite*_ ∕*A*_*Internal standard*_ = Δ*A*, where *A*_*metabolite*_ represents the Raman peak area imaging of a certain metabolite, *A*_*Internal standard*_ represents the Raman peak area imaging of the internal standard, and Δ*A* represents the constant obtained after the ratio. Statistical analysis of images obtained after ratio analysis for the semi-quantitative analysis of multiple intracellular metabolites.

We developed a workflow for the semi-quantitative analysis of intracellular symbiotic metabolism interactions based on CRM (Figure 1). A Raman spectra library was established to identify major cellular metabolites in the gills of *G. platifrons* with high abundance, including carbohydrates, amino acids, and sterols (Table S1).^26^ Compared to mammalian cells, the biochemical composition of bacteriocytes is more complex and heterogeneous. Moreover, symbionts containing nucleic acids are distributed in the cytoplasm, making it challenging to compartmentalize the cells because the characteristic peaks are unavailable. Hence, we employed a multivariate analysis strategy combining K-means clustering and true component analysis to identify molecules that can consider small differences in the peak positions and intensities of multiple Raman peaks, giving rise to a better recovery rate and accuracy (Figure 1B). True component analysis is a linear combination of all spectral datasets calculated using analytical algorithms to identify and visualize components.^27^^,^^28^^,^^29^ Compared to the traditional method, fine intracellular details, including the endosymbiont, cytoplasm, nucleus, and lipid droplets, can be visualized, as confirmed by the results of confocal microscopy with fluorescence staining (Figure S1).

Owing to the Raman scattering difference in the cross-section between molecules as well as the influence of environmental conditions and equipment performance, the Raman spectrum intensity cannot directly reflect the abundance of the measured components.^30^ Provided that the wavelengths used do not induce resonance effects of the metabolites of interest,^31^^,^^32^^,^^33^ we applied a Raman peak area imaging normalization protocol^30^ to estimate the abundance of biomolecules, by calculating the normalized peak area (peak area ratio) of the Raman spectrum (internal standard method) (*A*_*metabolite*_ ∕*A*_*Internal standard*_ = Δ*A*) for the gill cells (Figure 1C). The internal standard should meet the following criteria: uniform distribution and stable content. There is no gold-standard molecule available as an internal standard molecule in the cellular analysis as the distribution of most biomolecules was uneven or hypervariable. We found the Raman peak of 1,341 cm^−1^, which could be tentatively assigned to threonine (Tables S1 and S2), met the criteria. Based on the Raman characteristic peak area imaging of multiple metabolites, we found that threonine was relatively uniformly distributed in the cell, and further statistical analysis showed that threonine fluctuated less in the cell relative to other metabolites (Figures S2A and S2B). And combined with the metabolomics results, we found that its content did not change significantly relative to other metabolites even after de-colonization (Figure S2C). Thus, we attempted to select it as the internal standard. The protocol was tested on our mock sample (Figure S3). We mixed glucose of different concentrations with threonine of the same concentration, functioning as the internal standard, and found that the ratio of the Raman peak intensity of glucose to threonine and the concentration of threonine was linearly correlated (R^2^ = 0.9998, Figure S3). Our results confirmed that the methodology can give reliable quantity estimation at a subcellular scale. Thus, in the follow-up study, we used 1,341 cm^−1^ as the internal standard by univariate imaging to study the content of a variety of key metabolites *in situ* and in de-symbiotic gill cells.^34^^,^^35^^,^^36^ The quantitative findings were validated by metabolomics analyses and further cross-validated against scientific questions by electron microscopy ultrastructural data and genomics data.^37^^,^^38^

### High-resolution CRM for gill cells

**Figure 2:**
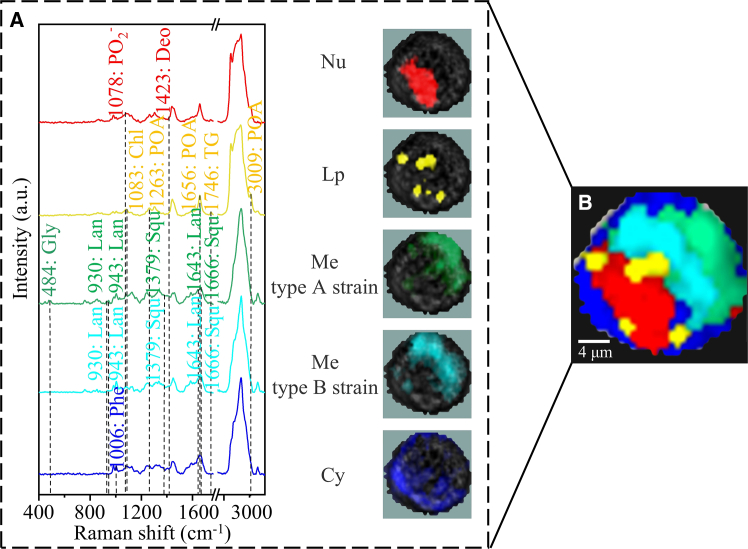
CRM imaging analysis of the gill cell *in situ* (A) Average Raman spectrum of nucleus (Nu), lipid droplets (Lp), symbionts (Me-type A strain, Me-type B strain), and cytoplasm (Cy) of bacteriocytes *in situ*. Deoxyribose, Deo; cholesterol, Chl; triglycerides, TG; palmitic acid, POA; glycogen, Gly; lanosterol, Lan; squalene, Squ; phenylalanine, Phe. (B) Raman imaging of bacteriocytes *in situ* showing the distribution of cellular components, including nucleus (red), lipid droplets (yellow), symbionts (green, cyan), and cytoplasm (blue). The white light image corresponding to Raman imaging images are shown in Figure S7.

Based on multivariate metabolic analysis, bacteriocytes *in situ* were clustered into five categories (Figure 2). The red cluster was located at the basal part of the cell, and featured Raman peaks were observed at approximately 1,078 cm^−1^, which corresponds to PO_2_^−^ stretching,^39^ and 1,423 cm^−1^ representing deoxyribose to display DNA,^40^^,^^41^ which was significantly stronger than other regions, suggesting that this region represented the nucleus. The yellow cluster was small dots near the nucleus, with feature signals at approximately 1,746 cm^−1^, which could be associated with vibrations of C=O^42^ representing triglycerides^43^^,^^44^ (Tables S1 and S2). These results suggest that the yellow-labeled regions were lipid droplets.^44^ Compared to other parts of the cell, the lipid droplets were characterized by strong bands at approximately 1,263, 1,656, and 3,009 cm^−1^, which could be attributed to palmitoleic acid (Tables S1 and S2), and at approximately 1,083 cm^−1^, representing cholesterol^31^ (Tables S1 and S2). The blue cluster filled the whole cell, with the feature band at approximately 1,006 cm^−1^ tentatively representing phenylalanine^40^^,^^45^ (Tables S1 and S2). The cluster was inferred to be the cytoplasm, which is commonly distributed in biological tissues. The green and cyan clysters were located on the opposite side of the nucleus, with significant signals tentatively attributed to lanosterol at approximately 930, 943, and 1,643 cm^−1^ (Tables S1 and S2 and Figure S4) and squalene at approximately 1,379 and 1,666 cm^-1^^46^^,^^47^^,^^48^ (Tables S1 and S2). The precursors of steroids, including squalene, are trademarks of type I methanotrophs.^49^ Therefore, these two clusters represent symbionts.

**Figure 3:**
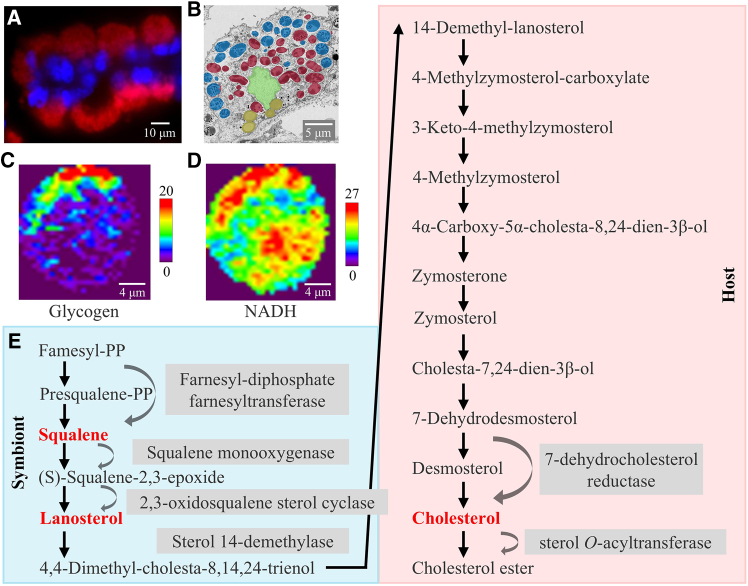
Analysis of microbial-mediated metabolic processes in the host intracellular compartment (A) Fluorescence *in situ* hybridization showing the location of the symbionts in the host cells. Red indicates symbionts; blue indicates the nucleus. (B) Scanning electron microscopic image of bacteriocyte *in situ*. The Me-type A strain (blue) containing electron-dense particles is distributed closer to the outer environment of the gill filaments; the Me-type B strain (red) is essentially devoid of electron-dense black particles and is distributed closer to the inner side. Further inward are the nucleus (green) and lipid droplets (yellow). (C) Glycogen was analyzed by integrating over a range of wave numbers at 484 ± 8 cm^−1^ in gill cell *in situ*.^42^^,^^51^^,^^52^ (D) NADH was analyzed by integrating over a range of wave numbers at 1,114 ± 8 cm^−1^^53^ in gill cell *in situ*. (E) The biosynthesis scheme of cholesterol in gill cells.^11^

Our results confirmed the polarized distribution of nuclei, as reported by Wang et al.^50^ 4’,6-diamidino-2-Phenylindole (DAPI) fluorescence signals were also detected in the nucleus and symbiont regions proposed by CRM, confirming the validity of the CRM analysis (Figures 3A and S1). A similar cellular distribution pattern was confirmed by transmission electron microscopy (TEM) (Figures 3B and S5A). The results confirmed that CRM is a reliable toolkit to discern bacteriocytes, which facilitates further comparison of possible metabolome changes during de-symbiosis. Using multivariate analysis, biomolecular profiles can be extracted and searched for biomolecular heterogeneity in different regions to offer us the opportunity to understand the metabolic processes in a cell without prior knowledge.

Recently, a growing body of research has focused on the genetic or metabolic divergence of endosymbiont strains among bacteriocytes.^54^ However, to the best of our knowledge, no studies have revealed possible metabolic divergence within a cell. Taking advantage of the sophisticated subcellular resolution and capacity for multiple metabolic pathway analysis offered by CRM, we identified that the symbionts could be subdivided into two phenotypic categories based on glycogen content, as indicated by a characteristic Raman band at approximately 484 cm^−1^. The symbionts dwelling in the outer region (Type A) exhibited higher glycogen concentrations (Tables S1 and S2; Figures 2A, 3C, and S6) than those elsewhere. This spatial distribution of glycogen reflects underlying differences in metabolic function.^42^^,^^51^^,^^52^ The symbionts are embedded in membrane chambers connected to the external environment through pathways^11^; therefore, resources including methane and oxygen tend to be richer in symbiont vesicles located adjacent to the water. Different metabolic strategies may be adopted by the methane-oxidizing bacteria in response to nutrient conditions. In the outer part adjacent to the nutrient-rich seawater, methanotrophic bacteria can produce a large amount of NADH by oxidizing methanol to formaldehyde and finally to carbon dioxide (Figures 3D and S6). Abundant NADH, methane, and oxygen may accelerate methane oxidation to methanol, leading to the accumulation of methanol.^55^ Methanol can promote the accumulation of glycogen granules in methane-oxidizing bacteria.^56^ Abundant hexose intermediates may also facilitate glycogen accumulation during sugar storage. The scanning electron microscopic imaging of the bacteriocytes confirmed the different metabolic status of the symbionts (Figure 3B).^37^ The tiny electron-dense granules aggregated in the outer part of the cell fit the characteristics of glycogen.^57^ Several studies have shown that the differentiation of microorganisms into subgroups with different functions in the same host may be key for their successful association with different environments.^54^ These results highlight the possibility that CRM can facilitate studies on how the symbiont is adapted to heterogeneous habitats in a cell and may be the mainstay of host organisms' perception of environmental change.

### Semi-quantitative Raman imaging of gill cells during the stages of “de-symbiont” at subcellular scale

**Figure 4:**
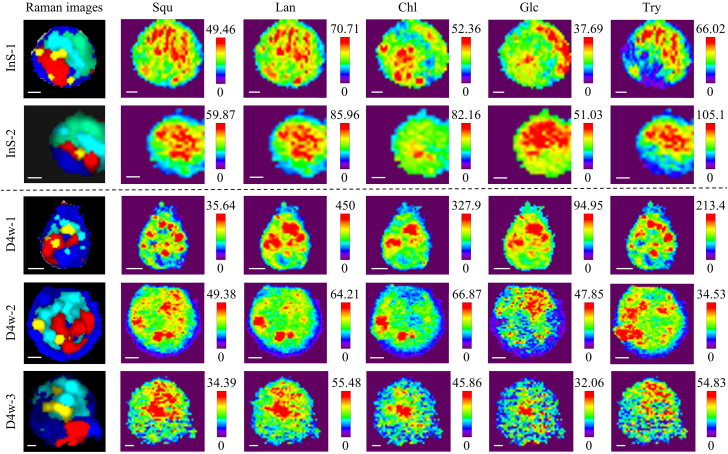
Raman imaging analysis of metabolite distribution in gill cells *in situ* (Ins) and after 4 weeks “de-symbiont” treatment (D4w) Raman imaging of gill cells, including gill cells *in situ* (Ins) and after 4 weeks “de-symbiont” treatment (D4w) shows the distribution of nucleus (red), lipid droplets (yellow), symbionts (green, cyan), and cytoplasm (blue). Scale bars: 3 μm. The white light images corresponding to Raman imaging images are shown in Figure S6. Univariate analysis was performed by integrating over a wavenumber range corresponding to relevant biomolecules: squalene (Squ) regions at around 1,666 ± 8 cm^−1^, lanosterol (Lan) regions at around 1,643 ± 8 cm^−1^, cholesterol (Chl) regions at around 1,083 ± 8 cm^−1^, glucose (Glc) regions at around 1,127 ± 8 cm^−1^, and tryptophan (Try) regions at around 1,557 ± 8 cm^−1^. The white light image corresponding to Raman imaging images are shown in Figure S7.

**Figure 5:**
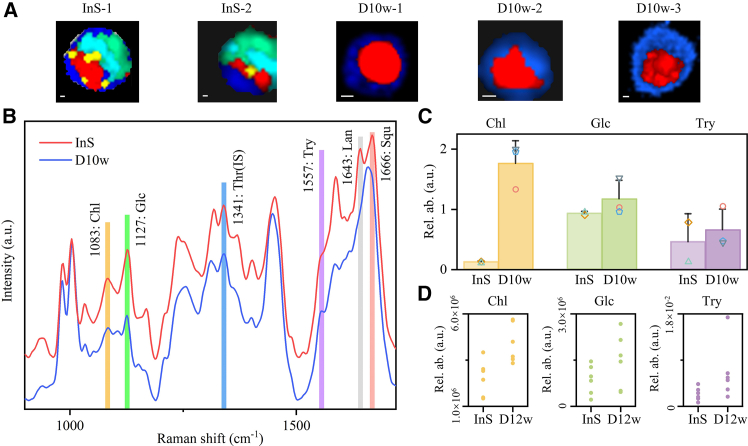
Semi-quantitative analysis of components of gill cells (A) Raman imaging of gill cells *in situ* (Ins) and after 10 weeks “de-symbiont” treatment (D10w) shows the distribution of nucleus (red), lipid droplets (yellow), symbionts (green, cyan), and cytoplasm (blue). Scale bars: 1 μm. The white light images corresponding to Raman imaging images are shown in Figure S6. (B) Average Raman spectra of bacteriocytes *in situ* (Ins) and gill cells after 10 weeks “de-symbiont” treatment (D10w). Cholesterol, Chl; glucose, Glc; threonine, Thr; tryptophan, Try; lanosterol, Lan; squalene, Squ. (C) Raman semi-quantitative analysis of cholesterol, glucose, and tryptophan in gill cells by statistical analysis of Raman images after internal calibration normalization (data given as the mean ± SD, *n* = 2 for *in situ* cells, *n* = 3 for de-symbiosis cells). (D) The relative changes of cholesterol, glucose, and tryptophan contents in InS group and D12w group as measured by metabolomics. The white light image corresponding to Raman imaging images are shown in Figure S7 (data given as the mean ± SD, *n* = 6 replicates).

On the basis of analysis of the gill cells, we further visualized bacteriocytes during the “de-symbiont” process, and to demonstrate the potential for semi-quantitative application of CRM, detailed semi-quantitative estimations of the key metabolites were conducted in the gill cells with threonine (1,341 cm^−1^) as an internal standard to study the *in situ* dynamic changes of intracellular metabolites in distribution and contents (Figures 4 and 5).

We found that mosaics with featured Raman bands for the symbionts (approximately 1,643 and 1,666 cm^−1^) and lipid droplets decreased gradually during aquaculture deprived of methane, and the volume of bacteriocytes also decreased due to the loss of symbionts (Figures 4, 5A, 5B, and S5). This suggests that the maintenance of the endosymbiont is dependent on a continuous supply of substrate and that a chronic lack of energy can lead to considerable symbiont loss.^58^ The TEM results confirmed that the symbionts were gradually digested by the lysosome, making the cell smaller (Figure S5).

In the *in situ* bacteriocytes, we found that signals of squalene and lanosterol peaked in the green and cyan regions where the symbionts aggregated, and cholesterol was mainly distributed in the lipid droplet near the nucleus in our Raman data (Figure 4). Based on our genomic evidence,^59^ the mussel host lacks multiple enzymes responsible for squalene synthesis, which yet can be encoded by the symbionts. Additionally, the mussel host is also massively transcribing sterol-related genes inside the bacteriocytes, where they acquire nutrients from symbionts (Figure 3E).^11^ It was hypothesized that the hosts and symbionts could cooperate to complete steroid biosynthesis by relay. The upstream and downstream components of cholesterol production are conducted by symbionts and hosts, respectively^11^ (Figure 3E). Combined with genomic data, our results probably provide the evidence for the association of metabolic interactions between hosts and symbionts (Figure 3E). Sterol precursors (squalene and lanosterol) may be synthesized with the assistance of methanotrophic symbionts and then recruited by the host. The vesicles are transported to the endoplasmic reticulum to form droplets and are integrated with enzymes to complete steroid biosynthesis.

During the 4 weeks of de-symbiosis, some cells had the highest concentration of squalene only in the region of the symbiotic bacteria and some had high concentrations in both the lipid droplets and the symbiont region, lanosterol had high concentrations in the lipid droplets and a portion of the symbiont region of the cells, and the highest cholesterol concentrations continued to be found in the lipid droplets (Figure 4). It is speculated that during de-symbiosis, the hosts, in addition to co-operating with the symbiont in the synthesis of cholesterol, may also be gradually digesting the additional nutrients obtained by the symbiont in order to maintain their ability to survive under conditions of chronic methane deprivation. Cholesterol increased after 10 weeks “de-symbiont” treatment (Figures 5C and S5), suggesting that the host may digest the symbionts and assimilate squalene to generate steroids as energy sources during nutrient-deprived conditions.

The glucose content within in situ bacteriocytes peaked in areas densely colonized by symbionts. In the fourth week of de-symbiosis, some cells had higher concentrations of glucose in the symbiont region, some in the lipid droplets, and others in both the symbiont and lipid droplets regions (Figure 4) and the content increased after 10 weeks of “de-symbiont” treatment (Figure 5C). Thus, our results suggest that without the supply of methane, the cell’s previously stored polysaccharides, such as glycogen, can be catabolized into glucose for energy production, or perhaps host digestion of the symbiont may have led to the hydrolysis of polysaccharides, as proposed by Ponnudurai et al.^12^ The content of amino acids, such as tryptophan, increased in response to methane starvation, suggesting that the host might actively synthesize it to meet the physiological needs (Figures 5C and 5D). The observed data indicate that microorganisms adapt their metabolic activities in response to alterations in their environment, prompting the host to adjust its own metabolic strategies accordingly. This dynamic interaction suggests that microorganisms have the potential to function as indicators of environmental change for the host organism. These findings are consistent with those of a metabolic study^60^ (Table S3), suggesting that our CRM toolkit not only provides an approach for studying host-symbiont metabolic interactions in deep-sea chemosynthetic ecosystems but also has the potential for broader applications in other symbiotic systems and diverse organisms. The semi-quantitative capability of CRM at subcellular resolution and the reliability of capturing metabolic signatures have been validated, indicating that it could be extended to study metabolic compartmentalization of extreme microorganisms, nutrient exchanges in cells or tissues, or even dynamic metabolite trafficking in eukaryotic organelles. By integrating 3D Raman scanning with depth-dependent signal correction,^61^ this method may further enhance accuracy of visualization of intracellular metabolite distribution and quantitative analysis of concentration within complex tissues or cells, offering a versatile tool for biology research.

### Limitations of the study

This method provides us with a way to investigate the metabolic processes in cells. However, in order to study the intracellular metabolic mechanism more precisely, we should increase the number of samples so as to investigate the differences and generalizations among cells. Metabolites of low concentration cannot be recovered in the current platform owing to the limited resolving power of the Raman methodology. The rapidly developing surface-enhanced Raman scattering technology will improve the sensitivity of our platform, making it possible to discriminate more metabolites. In addition, fixation treatments for samples in extreme deep-sea environments, such as cell fixation and *ex vivo* preservation operations, may introduce analytical bias in metabolic states and are difficult to meet the demands of online monitoring. In the future, how to detect deep-sea samples without the need for fixation and extraction is a key issue to be addressed.

## Resource availability

### Lead contact

Requests for further information and resources should be directed to and will be fulfilled by the lead contact, Xin Zhang (xzhang@qdio.ac.cn).

### Materials availability

This study did not generate new materials.

### Data and code availability

All the data reported in this paper will be shared by the lead contact upon request.

This paper does not report original codes.

Any additional information required to reanalyze the data reported in this paper is available from the lead contact upon request.

## Acknowledgments

This research was supported by the following grants: the 10.13039/501100001809National Natural Science Foundation of China (42327805, 42221005, 42030407, and 42506179), Marine S&T Fund of Shandong Province for 10.13039/501100010954Pilot National Laboratory for Marine Science and Technology (2022QNLM030004-3), 10.13039/501100002858China Postdoctoral Science Foundation under Grant (2024M751269), and 10.13039/501100007129Shandong Provincial Natural Science Foundation (ZR2024QD074).

## Author contributions

X.Z., M.W., W.H., and C.L. designed research; W.H., Z.Z., H.C., M.L., H.Z., and Y.W. performed research; S.X. and L.L. provided support for lab management and technical assistance; W.S. and Y.Z. provided data from the scanning electron microscope; W.H., M.W., X.Z., Z.Z., X.G., and H.C. analyzed data; W.H., M.W., and X.Z. wrote the paper; X.Z., C.L., and Z.L. supervised research.

## Declaration of interests

The authors declare no competing interests.

## STAR★Methods

### Key resources table

**Table:** 

REAGENT or RESOURCE	SOURCE	IDENTIFIER
**Biological samples**		

*Gigantidas platifrons*	Collected by authors	N/A

**Chemicals, peptides, and recombinant proteins**		

DL-3-Aminoisobutyric acid	Sigma-Aldrich	CAS 144-90-1
Palmitoleic acid	Sigma-Aldrich	CAS 373-49-9
sn-Glycerol 3-phosphate lithium salt	Sigma-Aldrich	CAS 17989-41-2
Ethanolamine	Sigma-Aldrich	CAS 141-43-5
Lanosterol	Sigma-Aldrich	CAS 79-63-0

**Software and algorithms**		

WITec Project plus	WITec alpha	300 R
GRAMS/AI software	Thermo Fisher Scientific	9.1

**Other**		

Confocal Raman Imaging	WITec alpha	300 R
Ultramicrotome	Leica	EMUC7
Transmission electron microscopy	HT7700	Hitachi
Confocal Microscopy System	ZEISS	LSM 900
Scanning electron microscope	FEI	Helios NanoLab 600i
Metabolomics data	https://doi.org/10.1016/j.scitotenv.2024.178048	N/A

### Experimental model and study participant details

This study was based on deep-sea mussel *Gigantidas platifrons*, and no experimental models were used.

### Method details

#### Sample collection and domestication

The deep-sea mussels *G. platifrons* were collected in the active cold seeps area (119°17′08.287″E; 22°06′55.425″N, temperature:3.56°C, salinity:34.63 psu) of the South China Sea during the “Kexue” cruise in 2020. After the samples were brought to the deck using a thermal-preserve sampler carried by the remotely operated vehicle (ROV) “Faxian”, some samples were quickly dissected for further processing, and the remaining samples were cultured in a pre-cooled seawater circulating water culture system. The circulating water system controls the temperature to 3.5±0.5°C and the salinity to 34.5±0.5 psu, and no methane was supplied during the subsequent breeding process, allowing the symbionts to escape the hosts to complete mussel de-symbiosis.

#### Cell extraction and preservation

Deep-sea mussel gill cells were obtained after 1 h incubation in 0.25%–1% trypsin (diluted in seawater) at 4°C. After filtering through a 40 μm membrane to remove the remaining cell debris, the cells were centrifuged at 1000 × *g* at 4°C for 5 min. The cells obtained were immersed in methanol and stored at -80°C for subsequent Raman detection. While methanol fixation preserves ultrastructural integrity and broadly retains cellular composition comparable to fresh samples, preliminary validation experiments revealed that specific metabolites exhibited spectral alterations (peak shifts or intensity variations). To evaluate potential spectral distortions induced by methanol fixation, paired analyses were performed on 13 days of de-symbiosis cells comparing fixed and unfixed samples. Significantly different peaks (e.g., 980 cm^-1^) were excluded from subsequent Raman peak attribution. Methanol fixation protocols were rigorously maintained across all experimental groups (symbiont-containing and de-symbiotic) to ensure analytical consistency.

#### Confocal Raman imaging

Raman spectra were collected and imaged using confocal Raman microspectroscopy (alpha 300R, WITec, Ulm, Germany) equipped with a laser operating at 532 nm and a 600 grooves/mm grating (UHTS 300, spectroscopic resolution 3 cm^-1^). Area scans were performed using ZEISS EC Epiplan (Carl ZEISS, Jena, Germany) 50×/0.75 and ZEISS EC Epiplan (Carl ZEISS, Jena, Germany) 100×/0.9. Before use, the system calibration was performed using a silicon wafer, which demonstrated a characteristic peak at 520 cm^-1^. The step size, total acquisition time, and laser power for each area of scan data acquisition were optimized for the optimal results. The x- and y-axis step sizes were approximately 450 nm, laser power was approximately 20 mW, and the integration time for the spectrum recording was 1 s. Depending on cell size, the total acquisition time for each sample ranged from 20 to 40 minutes. No degradation, charring, or significant displacement of the samples was observed during or after the measurements, as confirmed by bright-field inspection after imaging.

#### Raman data analysis and processing

For the acquisition of Raman spectral datasets, we combined multiple data-mining methods for analysis. Before feeding the data into these classification algorithms, the original dataset must be preprocessed. We used WITec Project plus software and GRAMS/AI software to perform baseline correction, cosmic ray removal (CRR), and smoothing (Savitzky-Golay) on the obtained dataset, followed by principal component analysis (PCA) to enhance the signal-to-noise ratio and necessary signal. K-means clustering and true component analysis were performed using WITec Project Plus software to image and obtain characteristic spectra of each cellular component. The color combination bitmap shows the intuitive distribution of components and cell morphology.

Based on multivariate analysis, the difference peaks of the characteristic spectra of different components were found for attribution and assigned according to the established library. Priority was given to the attribution of characteristic peaks that still had stable expression after methanol fixation, differed significantly in different culture stages or cell regions, and were closely related to the symbiotic state. The rest of the signals present may correspond to cellular universal components, so attribution was not performed for the time being. The most representative characteristic peaks of each component were selected, while the distribution of metabolites was indicated by the peak intensity imaging maps. The normalization principle was used to compare the differences between gill cells *in situ* and after the “de-symbiont” treatment by dividing the Raman peak intensity imaging of the selected internal standard (threonine) with various components to achieve semi-quantitative analysis. The final results are presented as heat maps. In addition, we quantified the specific numerical values of the metabolite changes using statistical analysis.

#### Validate imaging results with fluorescent staining

To verify the analysis results of the Raman imaging of the cell structure, we performed staining and Raman imaging on the same cell. We used 4’,6-diamidino-2-Phenylindole (DAPI) to fluorescently stain the nucleic acid in the gill cells. The nucleus could be observed as bright blue fluorescence, and the symbionts showed cloudy and weaker blue fluorescence. Subsequently, Raman scanning imaging was performed. To improve the staining effect, the cells were air-dried, stained, and air-dried, reducing cell volume.

#### Fluorescence *in situ* hybridization (FISH)

The dissected fresh gills were fixed overnight in pre-cooled 4% paraformaldehyde, rinsed three times with pre-cooled phosphate buffered saline (PBS), dehydrated in 75% ethanol, and stored at -20°C until use. The preserved gills were dehydrated using a series of low to high concentrations of ethanol (70, 80, 95, and 100%). The samples were then placed in xylene for 2 h until transparent, and then placed in paraffin at 60°C for 3 h. Embedded samples were sectioned using a microtome (Leica) with a thickness of 7 μm. The slices were first dewaxed with xylene and then rehydrated with a gradient of ethanol (100%, 95%, 80% and 70%) at reduced concentrations, and then washed twice with pure water after rehydration. After soaking the slices in 1xPBST for 5 min, hybridization solution containing probe Eub338 (5′-GCTGCCTCCCGTAGGAGT-3′) was added to the slices, then hybridized in a 46°C oven for 1 h, and washed twice with FISH washing solution after hybridization (10 min each), the nuclei were then stained with DAPI.

#### Transmission electron microscopy (TEM) observation

The dissected fresh gills were placed in a pre-cooled paraformaldehyde-glutaraldehyde (2%/2.5%) fixative solution. After fixation at 4°C for 24 h, the fixative was replaced once and then stored at 4°C. When used for TEM, the gills were removed from the fixative solution, rinsed twice with sterile seawater for 10 min each, fixed with 1.0% osmic acid for 1 h, and fixed samples were rinsed twice with ultrapure water. After the samples were dehydrated with gradient alcohol (30%, 50%, 70%, 95%, and 100%) three times (10 min each), dehydrated three times with 100% acetone (10 min each), and the samples were put into a mixed solution of 100% acetone and resin Epon812, finally the samples were embedded with pure resin Epon812. The embedding blocks were sectioned (EMUC7, Leica, Austria) and stained (3% acetate axis and 2% lead citrate), followed by TEM (HT7700, Hitachi, Japan) to observe the ultrastructure of the gill cells.

### Quantification and statistical analysis

Statistical analyses were performed using WITec Project Plus software with detailed statistical information for all experiments provided in respective figure legends and figures. Specifically, sample sizes included two biological replicates for *in situ* cell quantification, three for de-symbiosis cell analysis, and six independent replicates for metabolomics data. Results represent multiple independent experimental replicates, and reported values reflect the mean ± standard deviation unless otherwise noted.
