# Supplementary material for: In situ semi-quantitative imaging of intracellular metabolic interaction by confocal Raman microscopy
Source: iScience. 2025 Sep 12;28(10):113558. doi: 10.1016/j.isci.2025.113558 (PMC12508887; doi:10.1016/j.isci.2025.113558)
Supplement: Document S1. Figures S1–S7 and Tables S1–S3 [file mmc1.pdf]

iScience, Volume *28*

Supplemental information

In situ semi-quantitative imaging of intracellular metabolic interaction by confocal Raman microscopy

Wanying He, Minxiao Wang, Zhaoshan Zhong, Hao Chen, Shichuan Xi, Huan Zhang, Mengna Li, Wenhao Sun, Yan Zhang, Yun Wang, Xiaoxiao Guo, Lianfu Li, Zengfeng Du, Zhendong Luan, Chaolun Li, and Xin Zhang

Supplementary Information

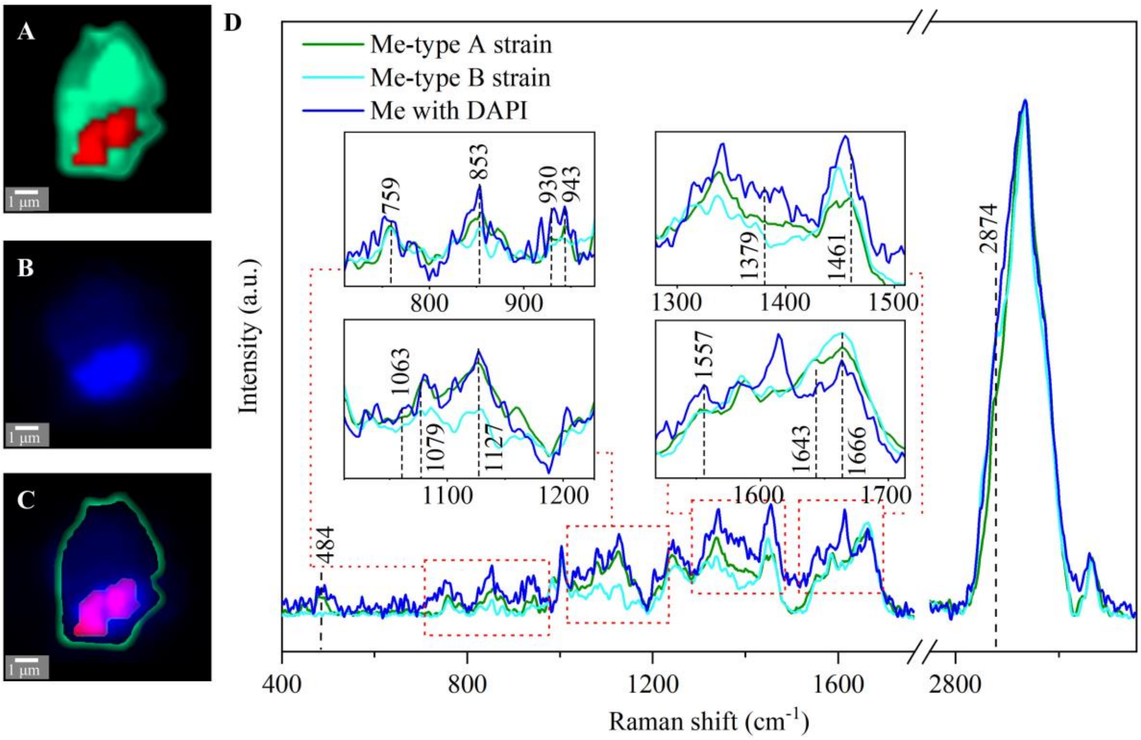

Fig S1. Fluorescence staining of gill cells bacteriocytes in situ.

(A) Raman imaging of bacteriocytes in situ, including symbionts (green) and nucleus (red). Scale bar: 1 μm. (B) Fluorescence staining image obtained by 4’,6-diamidino-2-phenylindole (DAPI), showing the distribution of nucleus and symbionts in the cells. Scale bar: 1 μm. (C) The results of (A) and (B) are superimposed, and the imaging results were consistent. Scale bar: 1 μm. (D) The average Raman spectra of symbionts with different metabolic functions (Me-type A strain and Me-type B strain) (Fig. 2) and symbionts by fluorescence staining (Me with DAPI). We verified the peaks of the characteristic metabolites of the symbionts, indicating lanosterol (Lan) (930, 943 and 1,643 cm^{-1}), squalene (Squ) (1,379 and 1,666 cm^{-1}) and glycogen (Gly) (484 cm^{-1}). In addition, it also contained tryptophan (Try) (759 and 1557 cm^{-1}), glutamine (Gln) (853 cm^{-1}), glucose (Glc) (1,063 and 1,127 cm^{-1}) and ethanolamine (Eth) (1,079, 1,461 and 2,874 cm^{-1}). Although DAPI could be used to stain cells, it destroyed the properties in situ of some components and affected the Raman peaks of some biomolecules [1]. For example, the Raman peaks in 1,614 cm^{-1} was different from the Raman peaks of unstained bacteriocytes.

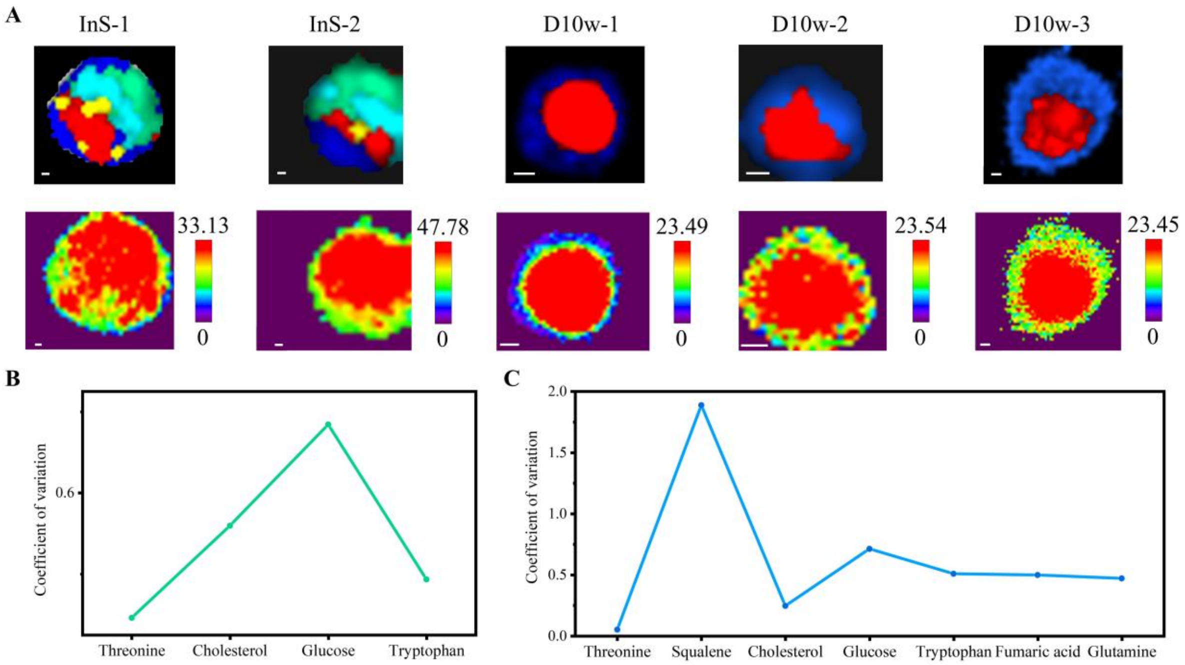

Fig S2. Selection of internal standard.

(A) CRM Integration images of gill cells, including bacteriocytes in situ (Ins) and cells after ten weeks "de-symbiont" treatment (D10w). Show the distribution of nucleus (red), lipid droplets (yellow), symbionts (green, cyan) and cytoplasm (blue). Scale bars: 1 μm. Threonine (Thr) analysis was performed by integrating over a wavenumber at around 1,341 ± 8 cm^{-1}, including bacteriocytes in situ (Ins) and cells after ten weeks "de-symbiont" treatment (D10w). (B) The mean coefficients of variation derived from the statistical analysis of Raman imaging data within the wave number ranges specific to threonine (1,341 ± 8 cm^{-1}), cholesterol (1,083 ± 8 cm^{-1}), glucose (1,127 ± 8 cm^{-1}), and tryptophan (1,557 ± 8 cm^{-1}) in the cells depicted in Figure a are provided here. (C) Coefficients of variation for metabolites in gill cells were obtained from metabolomics data in situ and after 7 days, 3 months and 1 year of "de-symbiosis" treatment.

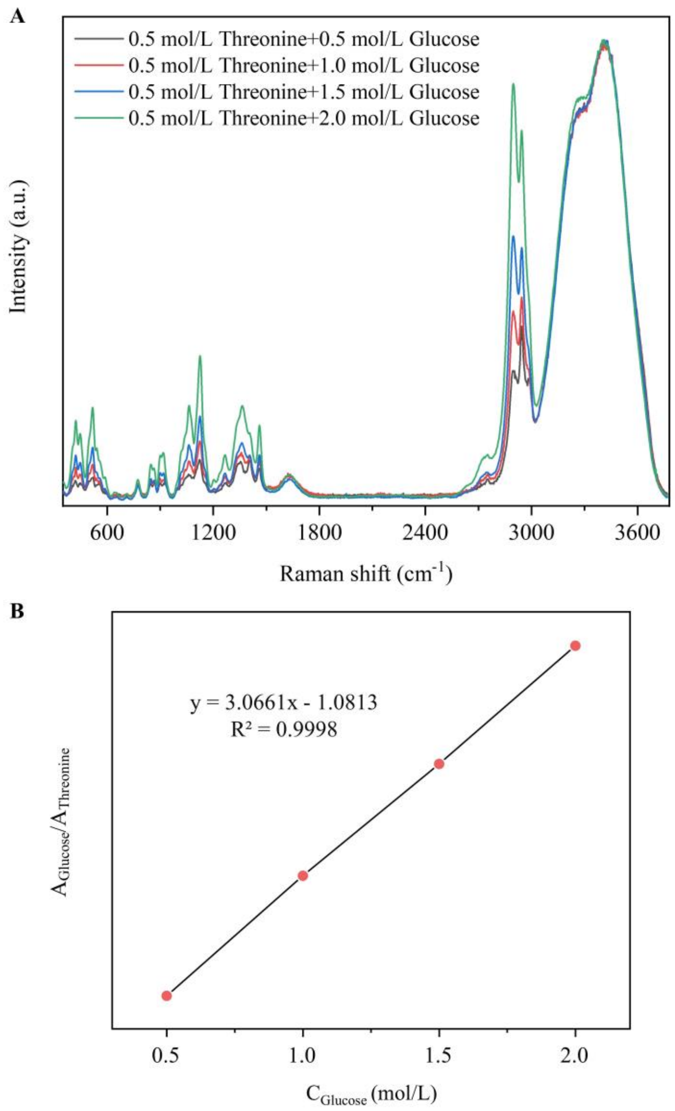

Fig S3. Quantification of glucose solutions by the internal standard method.

(A) Raman spectra of 0. 5 mol/L threonine solution (internal standard) mixed with 2.0 mol/L (green), 1.5 mol/L (blue), 1.0 mol/L (red), and 0.5 mol/L glucose (black). 1,341 cm^{-1} can be used as the characteristic peak of threonine and 1,126 cm^{-1} as the characteristic peak of lactic acid. When the abundance of threonine is constant, the peak intensity of the characteristic peak (1,341 cm^{-1}) of threonine remains basically unchanged, which can indicate that the peak intensity of the characteristic peak (1,126 cm^{-1}) of glucose increases with the increase of the abundance of threonine. Prove the reliability of threonine as an internal standard. (B) Raman semi-quantitative model of glucose using threonine solution as solvent.

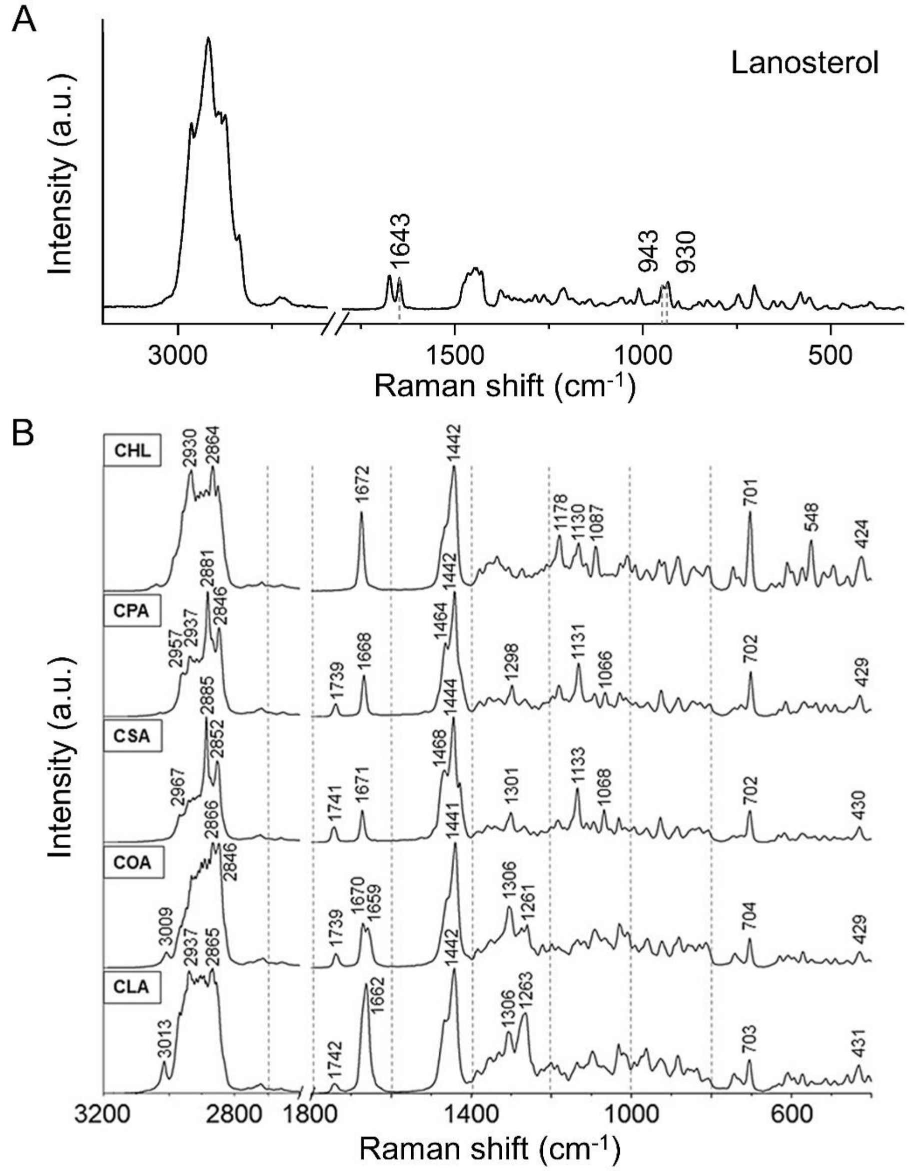

Figure S4. Raman spectra of the lanosterol, cholesterol (CHL) Cholesteryl palmitate (CPA), Cholesteryl stearate (CSA), Cholesteryl oleate (COA) and Cholesteryl linoleate (CLA) with the 532 nm excitation in the fingerprint region[2].

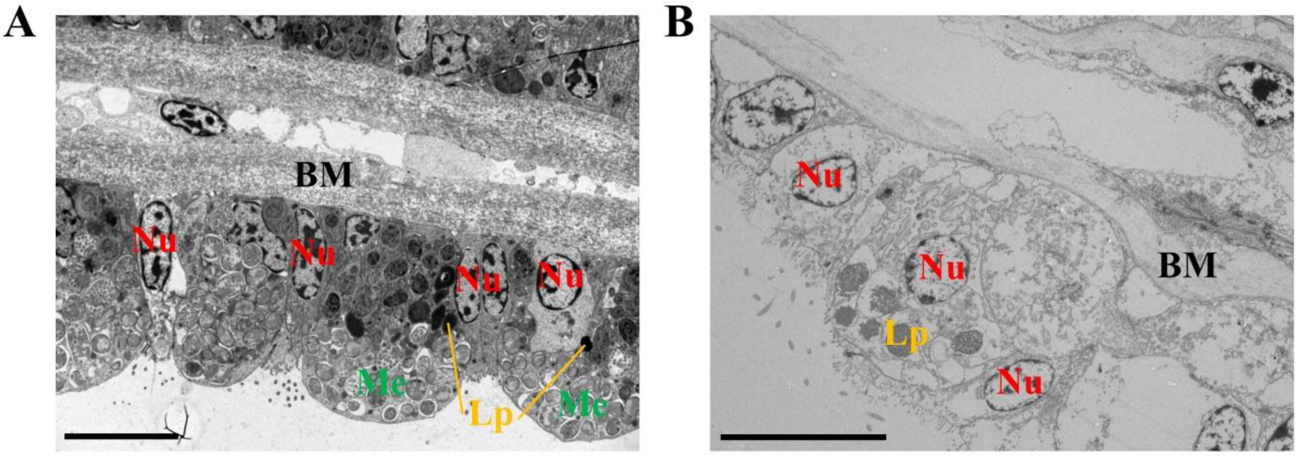

Fig S5. Transmission electron microscope images of gills of *G. platifrons*.

(A) Cellular morphology of the gills of *G. platifrons* in situ. (B) Cellular morphology of the gills of *G. platifrons* after "de-symbiont" treatment. Nu: nucleus; Lp: Lipid droplets; Me: Symbionts. Scale bars: 10 μm.

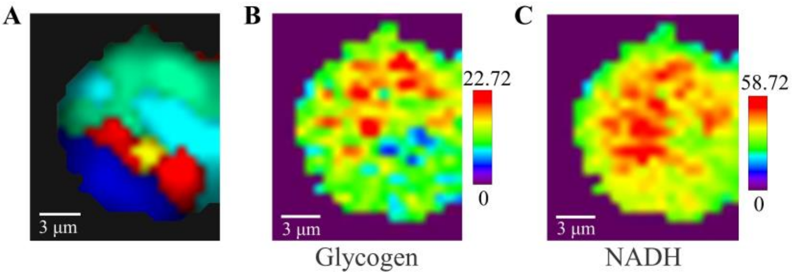

Fig S6. Analysis of microbial mediated metabolic processes in the host intracellular compartment.

(A) CRM Integration image of gill cells in situ, including nucleus (red), lipid droplets (yellow), symbionts (green, cyan) and cytoplasm (blue). (B) Raman intensity imaging of glycogen (484 cm^{-1})[3-5]. (C) Raman intensity imaging of NADH (1,114 cm^{-1})[6].

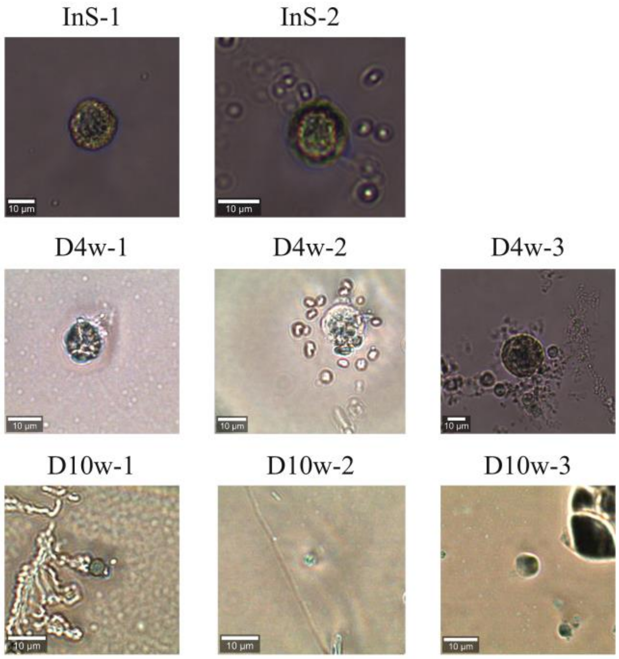

Fig S7. The white light images corresponding to the Raman imaging cells in situ (Ins), after four weeks (D4w) and ten weeks "de-symbiont" treatment (D10w).

S1Table. Raman spectra of metabolites in gill cells.

Metabolites	Raman bands (in cm^{-1})
D-Mannose solid (SCRC CSDS)	397(m), 475(m), 529(m), 575(mw), 665(m), 831(m), 845(m), 882(s), 914(m), 967(m), 1,018(mw), 1,079(s), 1,102(m), 1,135(m), 1208(w), 1,240(mw), 1,280(m), 1,312(m), 1,357(m), 1,458(s), 2,907(vs), 2,929(vs), 2,953(vs), 2,980(vs), 3,339(s), 3,500 (s)
D-Mannose solution (SCRC CSDS)	398(m), 486(m), 522(s), 576(m), 668(m), 832(m), 881(m), 914(mw), 963(mw), 1,066(m), 1,101(m), 1,139(m), 1,266(mw), 1,368(mw), 1,462(m), 2,944(vs)
sn-Glycerol 3-phosphate lithium salt solid (Sigma-Aldrich)	903(w), 1,528(w), 1,556 (w), 2,926(vw), 2,961(vw), 3,139(s), 3,497(m)
sn-Glycerol 3-phosphate lithium salt solution (Sigma-Aldrich)	404(w), 873(w), 981(s), 1,077(w), 1,092(w), 1,275(w), 1,394(w), 1,467(m), 1,644(m), 2,895(m), 2,963(m), 3,409(vs), 3,429(vs), 3,748(w)
Aspartic acid solid (Solarbio)	368(m), 465(m), 551(m), 748(m), 778(s), 872(ms), 901(s), 938(s), 1,084(s), 1,120(mw), 1,259(mw), 1,336(ms), 1,409(ms), 1,422(s), 1,507(mw), 1690(s), 2,956(vs), 2,997(ms), 3,013(s)
O-Phosphoryethano- lamine (Sigma-Aldrich)	357(mw), 418(s), 452(m), 503(mw), 544(s), 768(ms), 888(s), 918(ms), 946(s), 1,014(ms), 1,037(ms), 1,091(m), 1,186(m), 1,289(m), 1,332(m), 1,412(m), 1,451(s), 1,476(s), 1,545(mw), 1,629(mw), 2,120(mw), 2,573(w), 2,651(mw), 2,697(w), 2,748(m), 2,806(ms), 2,906(vs), 2,959(vs), 2,971(vs), 2,998(vs), 3,096(w)
L-Glutamine solid (Solarbio)	211(s), 454(w), 475(w), 543(mw), 624(mw), 778(mw), 853(ms), 895(m), 1000(w), 1,053(w), 1,098(m), 1,135(m), 1,167(m), 1,205(m), 1,285(m), 1,309(m), 1,332(ms), 1,356(w), 1,419(m), 1,450(m), 1,498(m), 1,550(w), 1,605(m), 1,646(w), 1,692(m), 2883(m), 2933(vs), 2960(vs), 2990(ms), 3213(m), 3409(m)
L-Glutamine solution (Solarbio)	853(w), 1,010(m), 1,102(m), 1,290(w), 1,352(w), 1,423(m), 1,634(m), 2,948(m), 3,280(vs), 3,410(vs)
D-Fructose solid (SCRC CSDS)	129(w), 248(w), 421(m), 464(mw), 525(m), 593(mw), 627(s), 781.32(w), 818(ms), 872(ms), 925(m), 978(m), 1,049(m), 1,082(ms), 1,142(m), 1,177(ms), 1,250(ms), 1,261(ms), 1,342(m), 1,397(mw), 1,471(s), 2,731(m), 2,902(s), 2,922(s), 2,940(vs), 2,962(vs), 2,991(vs), 3,015(vs), 3,357(mw), 3,399(m), 3,521(s)
D-Fructose solution (SCRC CSDS)	332(mw), 421(m), 457(mw), 521(m), 628(ms), 706(m), 784(w), 820(m), 870(m), 919(mw), 978(m), 1,084(m), 1,149(mw), 1,183(mw), 1,265(ms), 1,371(mw), 1,458(ms), 2,735(mw), 2,945(vs)
L-Threonine solid (Solarbio)	192(ms), 212(m), 335(mw), 443(mw), 490(mw), 564(s), 700(m), 748(mw), 775(m), 871(vs), 903(m), 931(ms), 1,043(m), 1,114(ms), 1,194(m), 1,252(m), 1,339(vs), 1,418(s), 1,465(m), 1,479(m), 1,548(m), 1,598(mw), 1,641(mw), 2,652(m), 2,750(m), 2,876(vs), 2,941(vs), 2,977(s), 2,997(ms), 3,021(vs), 3,162(mw)
L-Threonine solution (Solarbio)	146(mw), 555(mw), 662(w), 776(m), 870(mw), 932(mw), 1,041(w), 1,279(w), 1,345(s), 1,407(m), 1,461(m), 1,642(m), 2,754(mw), 2,945(ms), 2,988(ms), 3,379(vs), 3,412(vs), 3,423(vs)
N-Acetyl-L-Phenylalanine solid (Sigma-Aldrich)	112(vs), 242(ms), 342(mw), 384(mw), 419(w), 485(mw), 540(m), 566(mw), 621(m), 637(m), 702(w), 727(m), 828(m), 936(m), 976(ms), 1,006(s), 1,029(s), 1,043(m), 1,089(mw), 1,119(mw), 1,180(m), 1,198(ms), 1,275(m), 1,310(mw), 1,342(m), 1,380(mw), 1,436(m), 1,590(m), 1,605(s), 1,699(m), 2,848(m), 2,915(vs), 2,937(vs), 2,984(s), 3,015(m), 3,037(vs), 3,060(vs), 3,085(m), 3,172(m), 3,206(m), 3,331(s)
N-Acetyl-L-tryptophan solid	340(w), 429(w), 463(w), 502(w), 535(w), 574(w), 643(w), 693(m), 757(s), 806(w), 856(m), 965(w), 1,009(s),

(Sigma-Aldrich)	1,051(w), 1,121(m), 1,201(s), 1,235(w), 1,278(m), 1,334(s), 1,354(s), 1,423(s), 1,456(w), 1,486(w), 1,557(s), 1,574(s), 1,617(m), 1,707(m), 2,844(w), 2,913(s), 2,939(s), 2,967(m), 3,050(s), 3,216(m), 3,359(s), 3,416(s)
N-Acetyl-L-tryptophan solution (Sigma-Aldrich)	759(m), 878(m), 1,009(ms), 1,557(ms)
D-Glucose solid (SCRC CSDS)	419(ms), 513(ms), 563(m), 857(ms), 923(ms), 1,027(m), 1,074(m), 1,127(ms), 1,157(m), 1,229(mw), 1,266(mw), 1,333(m), 1,361(mw), 1,3890(mw), 1,433(w), 1,456(m), 2647(w), 2,725(mw), 2,767(mw), 2,885(s), 2,903(vs), 2,938(vs), 2,966(m), 2,974(ms), 2,992(vs), 3,269(m), 3,319(m), 3,394(mw), 3,438(mw)
D-Glucose solution (SCRC CSDS)	444(mw), 517(mw), 1,063(mw), 1,127(m), 1,372(w), 1,462(w), 1,641(m), 2,899(mw), 2,952(mw), 3,412(vs)
Lactic acid (SCRC CSDS)	7743(m), 796(mw), 825(ms), 870(m), 925(m), 1,048(m), 1,087(m), 1,133(mw), 1,458(m), 1,555(w), 1,725(m), 2,331(m), 2,728(mw), 2,736(mw), 2,947(vs), 2,999(s), 3,466(w), 3,481(w), 3,587(w), 3,719(w)
Lanosterol solid (Sigma-Aldrich)	395(mw), 466(mw), 558(m), 581(m), 631(mw), 652(mw), 704(m), 746(m), 797(mw), 828(mw), 851(mw), 907(mw), 934(ms), 947(ms), 1,010(m), 1,057(mw), 1,143(mw), 1,212(m), 1,263(mw), 1,286(mw), 1,378(m), 1,447(ms), 1,641(ms), 1,674(ms), 2,728(m), 2,877(vs), 2,919(vs), 2,963(vs)
Ethanolamine (Sigma-Aldrich)	482(m), 846(m), 873(ms), 1,032(mw), 1,081(m), 1,173(mw), 1,262(mw), 1,308(m), 1,358(m), 1,461(ms), 1,602(m), 2,779(mw), 2,874(vs), 2,934(vs), 3,191(mw), 3,306(vs), 3,366(ms)
Palmitoleic acid (Sigma-Aldrich)	98(m), 139(w), 214(ms), 319(mw), 890(m), 908(ms), 977(w), 1,063(m), 1,094(m), 1,116(mw), 1,263(m), 1,297(m), 1,442(ms), 1,656(s), 2,729(m), 2,855(vs), 2,882(vs), 2,903(vs), 2,930(vs), 3,009(ms)
DL-3-Aminoisobutyric acid (Sigma-Aldrich)	296(w), 316(m), 446(s), 507(m), 538(m), 567,626(s), 774(m), 837(vs), 891(s), 921(s), 1,003(m), 1,035(m), 1,053(m), 1,109(s), 1,135(m), 1,268(s), 1,313(vs), 1,331(s), 1,368(w), 1,396(w), 1,445(w), 1,466(s), 1,547(w), 1,582(m), 1,616(m), 1,689(m), 2,885(vs), 2,908(vs), 2,950(vs), 2,977 (vs), 2,993(vs)
Cholesterol [2, 7]	424, 548, 701, 1,087, 1,130, 1,178, 1,442, 1,672, 2,864, 2,930
Linoleic acid [2] (Sigma-Aldrich)	872, 904, 972, 1,075, 1,087, 1,107, 1,262, 1,300, 1,438, 1,654, 2,845, 2,885, 2,929, 3,002
Stearic acid [2]	896, 915, 984, 1,067, 1,105, 1,133, 1,178, 1,300, 1,409, 1,445, 1,466, 2,848, 2,882, 2,928
Squalene [8]	454(m), 804(m), 1,003(ms), 1,281(m), 1,330(ms), 1,382(s), 1,451(ms), 1,668(vs), 2,913(vs)
Oleic acid [2]	866, 976, 1,084, 1,120, 1,265, 1,306, 1,444, 1,657, 2,852, 2,891, 2,920, 3,004
Palmitic acid [2]	674, 898, 912, 980, 1,034, 1,067, 1,103, 1,132, 1,179, 1,426, 1,443, 1,467, 2,848, 2,881, 2,925, 2,967
Glycogen [5]	307, 326, 363, 406, 430, 446, 484, 938, 1,084, 1,130, 1,262, 1,337, 1,387, 1,460, 2,908
Glycerol [9]	1,049(m), 1,081(m), 1,109(w), 1,207(w), 1,254(m), 1,311(m), 1,357(s), 1,464(w), 2,752(s), 2,887(s), 2,914(m), 2,949 (m), 3,240(vs), 3,340(vs), 3,440(vs)
Glutamic acid solid [10]	623(m), 669(m), 750(w), 781(vw), 873(vs), 917(s), 988(m), 1,008(w), 1,042(vw), 1,080(m), 1,164(w), 1,182(m), 1,275(vw), 1,287(vw), 1,319(m), 1,346(w), 1,379(m), 1,422(m), 1,462(m), 1,637(m), 1,682(m)
Glutamic acid solution [10]	871(vw), 915(vw), 1,079(vw), 1,419(vw)

Serine solid [10]	610(m), 805(m), 814(m), 854(s), 922(w), 969(m), 1,010(s), 1,127(m), 1,220(m), 1,301(m), 1,327(s), 1,417(m), 1,464(m), 1,630(w)
Serine solution [10]	811(w), 857(m), 918(w,br), 976(w), 1,056(w,br), 1,089(w), 1,239(w,br), 1,348(m), 1,413(m), 1,469(m)
Valine solid [10]	430(w), 542(s), 665(m), 715(w), 753(m), 776(s), 825(m), 850(s), 902(w), 948(s), 965(m), 1,035(w), 1,066(m), 1,126(m), 1,144(w), 1,179(vw), 1,192(m), 1,273(m), 1,331(m), 1,354(m), 1,396(m), 1,427(vw), 1,454(m), 1,467(m,sh), 1,509(vw)
Valine solution [10]	757(m), 829(m), 847(vw), 948(m), 967(vw), 1,064(vw), 1,125(vw), 1,137(vw), 1,270(w,br), 1,328(m), 1,336(m), 1,362(m), 1,411(y), 1,450(w), 1,473(w)
Cystine solid [10]	455(m), 499(vs), 542(m), 613(m), 678(m), 785(s), 844(w), 873(m), 967(m), 1,041(vw), 1,092(w), 1,134(w), 1,196(w), 1,301(w), 1,341(s), 1,385(m), 1,410(m), 1,488(w), 1,625(w)
Cystine solution [10]	499(m), 678(vw), 787(vw), 1,343(vw)
Uric Acid [11]	385(m), 472(m), 503(m), 562(m), 627(vs), 659(w), 707(m), 784(m), 885(m), 999(s), 1,039(vs), 1,122(m), 1,234(m), 1,288(m), 1,356(w), 1,406(s), 1,499(m)， 1,595(m), 1,652(s), 1,684(w)
Taurine [12]	233, 326, 367, 472, 477, 523, 532, 592, 737, 849, 895, 964, 971, 1,033, 1,050, 1,109, 1,179, 1,220, 1,250, 1,257, 1,304, 1,343, 1,427, 1,459, 1,518, 1,597, 1,615, 2,915, 2,952, 2,987, 3,022
Adenine [13, 14]	536(m), 560(w), 623(m), 723(s), 898(w), 941(m), 1,024(w), 1,125(m), 1,162(w), 1,134(w), 1,248(m), 1,307(w), 1,332(m), 1,371(w), 1,419(w), 1,462(w), 1,482(m), 1,612(w),
Cytosine [13]	402(mw), 444(w), 537(m), 548(m), 568(w), 599(m), 792(s), 971(mw), 991(w), 1,011(w), 1,108(mw), 1,250(m), 1,275(s), 1,362(mw), 1,460(w), 1,493(w), 1,533(w), 1,653(mw), 1,690(w)
Guanine [13]	397(m), 496(m), 548(w), 563(m), 650(s), 711(w), 849(w), 937(m), 1,048(w), 1,158(w), 1,187(w), 1,234(m), 1,266(m), 1,360(m), 1,391(m), 14,22(m), 1,468(w), 1,479(w), 1,550(m), 1,602(w), 1,674(w)
Thymine [13]	429(m), 479(m), 561(m), 617(s), 740(s), 767(w,sh), 804(m), 984(m), 1,156(w), 1,216(w), 1,247(w), 1,261(w), 1,369(vs), 1,408(w), 1,435(w), 1,459(w), 1,490(m), 1,655(w,sh), 1,671(vs), 1,702(w)
Uracil [13, 14]	429(w), 529(w), 537(w), 556(m), 577(m), 790(vs), 984(w), 995(w), 1,100(w), 1,235(s), 1,255(w), 1,394(m), 1,418(m), 1,455(w), 1,504(w), 1,608(w), 1,646(m), 1,711(w)
Triglycerides [15, 16] (fatty acids)	1,073, 1,442, 1,657, 1,745
NADH [6]	391(w), 410(w), 716(m), 1087(w), 1,114(m), 1,172(w), 1,198(w), 1,245(m), 1,303(m), 1,324(w), 1,340(w), 1,375(m), 1,415(m), 1,510(w), 1,575(m), 1,600(w), 1,616(m), 1,681(s)
NADH in solution [6]	390(w), 526(m), 730(m), 830(w), 918(w), 940(w), 998(w), 1,084(m), 1,112(m), 1,182(w), 1,246(w), 1,308(m), 1,338(m), 1,378(m), 1,422(s), 1,458(w), 1,546(s), 1,578(w), 1,618(m), 1,688(s)
NAD^+ [17]	564(w,br), 642(w,br), 730(m), 834(w), 854(w), 888(w), 914(w), 1,032(vs), 1,084(w), 1,116(w), 1,186(w,br), 1,224(w), 1,254(w), 1,308(m), 1,338(s), 1,378(m), 1,422(w), 1,458(w), 1,484(w), 1,510(w), 1,580(m)

Abbreviations: w, weak; m, medium; s, strong; mw, medium weak; ms, medium strong; vs, very strong; sh, shoulder; br, broad.

S2 Table. Bands assignments for Raman spectra of metabolites in gill cells.

Peak Center (cm^{-1})	Molecular Vibration	Assignment	Location
~484	Skeletal modes, β(CCC) [5]	Glycogen [3-5]	Symbionts (type A)
~759	The ring breathing vibrations of indole ring [3-5]	Tryptophan (*SI Appendix*, Table S1)	Symbionts
~786	O-P-O, cytosine, uracil, thymine [3]	DNA [3, 4]	Nucleus
~853	Deformation mode of amino groups [18]	Glutamine (*SI Appendix*, Table S1)	Symbionts
~902	C-C-N [18]	Glutamine (*SI Appendix*, Table S1)	Symbionts
~930	-	Lanosterol (*SI Appendix*, Table S1)	Symbionts
~943	-	Lanosterol (*SI Appendix*, Table S1)	Symbionts
~1,006	ν_s(C-C) [14], symmetric ring breathing [19]	Phenylalanine [14, 19] (*SI Appendix*, Table S1)	Cytoplasm
~1,049	ν(C-O) or ν(C- N) [14]	Protein [14]	Cytoplasm (ciliated cells without bacteria)
~1,063	ν(CC), ν(CO), β(COH) [5]	Glucose (*SI Appendix*, Table S1)	Symbionts
~1,078	$\nu_s(PO_2^-)$ [20]	DNA [20]	Nucleus
~1,083	ν(C-C) [2]	Cholesterol [2]	Symbionts
~1,127	ν(CC), ν(CO), β(COH) [5]	Glucose (*SI Appendix*, Table S1)	Symbionts
~1,263	δ(=CH) [2]	Palmitoleic acid (*SI Appendix*, Table S1)	Lipid droplets
~1,297	$\tau(CH_2)$[2]	Palmitoleic acid (*SI Appendix*, Table S1)	Lipid droplets
~1,337	-	Tryptophan (*SI Appendix*, Table S1)	Symbionts
~1,341	δ(CH) [10]	Threonine (*SI Appendix*, Fig. S3)	Cell
~1,379	ν(C-C) [21]	Squalene [8, 21, 22]	Symbionts
~1,423	-	Deoxyribose [14, 23]	Nucleus
~1,442	$\alpha(CH_2/CH_3)$[2]	Cholesterol [2] or Palmitoleic acid (*SI Appendix*, Table S1)	Symbionts or Lipid droplets
~1,447	-	Lanosterol (*SI Appendix*, Table S1)	Symbionts
~1,461	$\beta(CH_2/CH_3)$ [2]	Ethanolamine (*SI Appendix*, Table S1)	Symbionts
~1,557	Indole ring stretching [10]	Tryptophan (*SI Appendix*, Table S1)	Symbionts
~1,643	-	Lanosterol (*SI Appendix*, Table S1)	Symbionts
~1,656	ν(C = C) [2]	Palmitoleic acid (*SI Appendix*, Table S1)	Lipid droplets
~1,666	ν(C=C) [21]	Squalene [8, 21, 22]	Symbionts
~1,746	ν(C = O) [2]	Triglycerides [15, 24]	Lipid droplets
~3,009	ν(=CH) [2]	Palmitoleic acid (*SI Appendix*, Table S1)	Lipid droplets

α, scissoring; β, bending; δ, deformation; τ, twisting; ν, stretching (s, symmetric; as, asymmetric).

S3 Table. Metabolomics data of the *G.platifrons* gills.

	LC-MS positive mode				LC-MS negative mode				GC-MS	
**Metabolites**	Squalene	Threonine	Tryptophan	**Metabolites**	Glutamine	Malic acid	Fumaric acid	**Metabolites**	Glucose	Cholesterol
**m/z**	411.39851	120.06545	205.09726	**m/z**	145.06046	133.01274	115.00209			
**InS#1**	0.0315638	0.0183705	0.0018296	**InS#1**	1.1706584	3.4236512	0.3245265	**InS#1**	969907	2436723
**InS#2**	0.0068331	0.0069148	0.0032015	**InS#2**	0.9803063	3.7413767	0.3879895	**InS#2**	829922	3918955
**InS#3**	0.0251428	0.0078045	0.0015041	**InS#3**	0.6768572	2.4963111	0.24252	**InS#3**	437546	3013165
**InS#4**	0.0107833	0.0120221	0.0043267	**InS#4**	0.6534882	1.608669	0.2236085	**InS#4**	1457196	1478790
**InS#5**	0.0060686	0.0088487	0.0025735	**InS#5**	1.2239749	3.3854754	0.3161007	**InS#5**	1271624	2911050
**InS#6**	0.0033424	0.0058252	0.0007417	**InS#6**	0.4755344	1.3710433	0.1846722	**InS#6**	216178	1425073
**G7D#1**	0	0.0193246	0.014926	**G7D#1**	0.8512116	10.806096	0.7588787	**G7D#1**	2384357	2473976
**G7D#2**	0.000486	0.0127264	0.0053472	**G7D#2**	0.8243041	14.132578	0.8488853	**G7D#2**	1854635	2809081
**G7D#3**	0	0.0144467	0.0036008	**G7D#3**	0.5598814	9.0266255	0.6638025	**G7D#3**	1428155	2664255
**G7D#4**	0.000371	0.015208	0.0040573	**G7D#4**	0.5579879	8.5862434	0.5600669	**G7D#4**	1219182	4450823
**G7D#5**	0.0010903	0.0156442	0.0124378	**G7D#5**	1.308238	15.940457	1.0521884	**G7D#5**	3405827	3541922
**G7D#6**	0	0.0179659	0.0179882	**G7D#6**	0.442522	13.595973	0.7849709	**G7D#6**	2499390	4346945
**G3M#1**	0.0009896	0.0278701	0.0032904	**G3M#1**	1.3283239	12.940491	0.8443779	**G3M#1**	1450349	3691427
**G3M#2**	0	0.0124996	0.0064356	**G3M#2**	0.342854	22.093607	1.322782	**G3M#2**	2153253	3546111
**G3M#3**	0	0.009618	0.0050925	**G3M#3**	1.1941617	18.709953	1.2702518	**G3M#3**	1651952	5693870
**G3M#4**	0	0.0129616	0.0055942	**G3M#4**	1.0800916	12.472268	1.0056855	**G3M#4**	2673655	3333892
**G3M#5**	0.0007481	0.0093458	0.0018751	**G3M#5**	0.2312344	9.2415777	0.6642346	**G3M#5**	502765	4493835
**G3M#6**	0	0.0155345	0.0173278	**G3M#6**	0.5207209	7.6552304	0.5478277	**G3M#6**	465030	5635250
**G1Y#1**	0	0.0172245	0.0046115	**G1Y#1**	0.3099973	6.8674523	0.5215115	**G1Y#1**	360579	5389318
**G1Y#2**	0	0.0130055	0.0024706	**G1Y#2**	0.346078	9.5943873	0.6876368	**G1Y#3**	136025	3348213
**G1Y#3**	0	0.0075671	0.0019392	**G1Y#3**	0.1402449	3.0809638	0.3248785	**G1Y#4**	72910	2580749
**G1Y#4**	0	0.0091701	0.0013651	**G1Y#4**	0.0858833	2.0400499	0.2152543	**G1Y#5**	239210	5845566
**G1Y#5**	0	0.0098672	0.0073135	**G1Y#5**	0.1646239	6.1404182	0.5790117	**G1Y#6**	179722	4787146
**G1Y#6**	0	0.0152338	0.0016819	**G1Y#6**	0.1345238	2.632249	0.318189			

Supplementary Information References

1. Wang, S., An, Z., and Wang, Z. (2020). Bioconversion of methane to chemicals and fuels by methane-oxidizing bacteria. Advances in Bioenergy *5*, 169-247. https://doi.org/10.1016/bs.aibe.2020.04.005.
2. Czamara, K., Majzner, K., Pacia, M.Z., Kochan, K., Kaczor, A., and Baranska, M. (2015). Raman spectroscopy of lipids: a review. J. Raman Spectrosc. *46*, 4-20. https://doi.org/10.1002/jrs.4607.
3. Stone, N., Kendall, C., Smith, J., Crow, P., and Barr, H. (2004). Raman spectroscopy for identification of epithelial cancers. Faraday Discuss. *126*, 141. https://doi.org/10.1039/b304992b.
4. Stone, N., Kendall, C., Shepherd, N., Crow, P., and Barr, H. (2002). Near-infrared Raman spectroscopy for the classification of epithelial pre-cancers and cancers. J. Raman Spectrosc. *33*, 564-573. https://doi.org/10.1002/jrs.882.
5. Wiercigroch, E., Szafraniec, E., Czamara, K., Pacia, M.Z., Majzner, K., Kochan, K., Agnieszka, K., Baranska, M., and Malek, K. (2017). Raman and infrared spectroscopy of carbohydrates: A review. Spectrochimica Acta Part A: Molecular and Biomolecular Spectroscopy *185*, 317-335. https://doi.org/10.1016/j.saa.2017.05.045.
6. Chen, D., Yue, K.T., Martin, C., Rhee, K.W., Sloan, D., and Callender, R. (1987). Classical Raman spectroscopic studies of NADH and NAD^+ bound to liver alcohol dehydrogenase by difference techniques. Biochemistry-Us *26*, 4776-4784. https://doi.org/10.1021/bi00389a027.
7. Hanlon, E.B., Manoharan, R.Y., Koo, T.W., Shafer, K.E., T, M.J., M, F., R, K.J., Itzkant I, R, D.R., and Feld, M.S. (2000). Prospects for in vivo Raman spectroscopy. Phys. Med. Biol. *45*, R1-R59. https://doi.org/10.1088/0031-9155/45/2/201.
8. Chun, H.J., Weiss, T.L., Devarenne, T.P., and Laane, J. (2013). Vibrational spectra and DFT calculations of squalene. J. Mol. Struct. *1032*, 203-206. https://doi.org/10.1016/j.molstruc.2012.10.008.
9. Mudalige, A., and Pemberton, J.E. (2007). Raman spectroscopy of glycerol/D_2O solutions. Vib. Spectrosc. *45*, 27-35. https://doi.org/10.1016/j.vibspec.2007.04.002.
10. Zhu, G., Zhu, X., Fan, Q., and Wan, X. (2011). Raman spectra of amino acids and their aqueous solutions. Spectrochimica Acta Part A: Molecular and Biomolecular Spectroscopy *78*, 1187-1195. https://doi.org/10.1016/j.saa.2010.12.079.
11. Kodati, V.R., Tu, A.T., and Turumin, J.L. (1990). Raman spectroscopic identification of uric-acid-type kidney stone. Appl. Spectrosc. *44*, 1134-1136. https://doi.org/10.1366/0003702904086470.
12. Freire, P.T.C., Melo, F.E.A., and Filho, J.M. (1996). Polarized Raman and infrared spectra of taurine crystals. J. Raman Spectrosc. *27*, 507-512. https://doi.org/10.1002/(SICI)1097-4555(199607)27:7<507::AID-JRS987>3.0.CO;2-3.

13. De Gelder, J., De Gussem, K., Vandenabeele, P., and Moens, L. (2007). Reference database of Raman spectra of biological molecules. J. Raman Spectrosc. *38*, 1133-1147. https://doi.org/10.1002/jrs.1734.

14. Chan, J.W., Taylor, D.S., Zwerdling, T., Lane, S.M., Ihara, K., and Huser, T. (2006). Micro-Raman spectroscopy detects individual neoplastic and normal hematopoietic cells. Biophysical Journal *90*, 648-656. https://doi.org/10.1529/biophysj.105.066761.

15. Silveira, L., Sathaiah, S., Zângaro, R.A., Pacheco, M.T.T., Chavantes, M.C., and Pasqualucci, C.A.G. (2002). Correlation between near-infrared Raman spectroscopy and the histopathological analysis of atherosclerosis in human coronary arteries. Laser. Surg. Med. *30*, 290-297. https://doi.org/10.1002/lsm.10053.

16. Rava, R.P., Baraga, J.J., and Feld, M.S. (1991). Near infrared Fourier transform Raman spectroscopy of human artery. Spectrochimica Acta Part A: Molecular and Biomolecular Spectroscopy *47*, 509-512. https://doi.org/10.1016/0584-8539(91)80129-7.

17. Yue, K.T., Martin, C.L., Chen, D., Nelson, P., Sloan, D.L., and Callender, R. (1986). Raman spectroscopy of oxidized and reduced nicotinamide adenine dinucleotides. Biochemistry-Us *25*, 4941-4947. https://doi.org/10.1021/bi00365a033.

18. Monfared, A.M.T., Tiwari, V.S., Trudeau, V.L., and Anis, H. (2015). Surface-enhanced Raman scattering spectroscopy for the detection of glutamate and γ-aminobutyric acid in serum by partial least squares analysis. Ieee Photonics J. *7*, 1-16. https://doi.org/10.1109/JPHOT.2015.2423284.

19. Bonnier, F., and Byrne, H.J. (2012). Understanding the molecular information contained in principal component analysis of vibrational spectra of biological systems. Analyst *137*, 322-332. https://doi.org/10.1039/c1an15821j.

20. Ponkumar, S., Duraisamy, P., and Iyandurai, N. (2011). Structural analysis of DNA interactions with magnesium ion studied by Raman spectroscopy. American journal of biochemistry and biotechnology *7*, 135-140. https://doi.org/10.3844/ajbbsp.2011.135.140.

21. Ishitsuka, K., Koide, M., Yoshida, M., Segawa, H., Leproux, P., Couderc, V., Watanabe, M.M., and Kano, H. (2017). Identification of intracellular squalene in living algae, *Aurantiochytrium mangrovei* with hyper-spectral coherent anti-Stokes Raman microscopy using a sub-nanosecond supercontinuum laser source. J. Raman Spectrosc. *48*, 8-15. https://doi.org/10.1002/jrs.4979.

22. Hall, D.W., Marshall, S.N., Gordon, K.C., and Killeen, D.P. (2016). Rapid quantitative determination of squalene in shark liver oils by Raman and IR spectroscopy. Lipids *51*, 139-147. https://doi.org/10.1007/s11745-015-4097-6.

23. Ruiz-Chica, A.J., Medina, M.A., Sánchez-Jiménez, F., and Ramírez, F.J. (2004).

Characterization by Raman spectroscopy of conformational changes on guanine-cytosine and adenine-thymine oligonucleotides induced by aminooxy analogues of spermidine. J. Raman Spectrosc. *35*, 93-100. https://doi.org/10.1002/jrs.1107.

24. El-Mashtoly, S.F., Niedieker, D., Petersen, D., Krauss, S.D., Freier, E., Maghnouj, A., Mosig, A., Hahn, S., Kötting, C., and Gerwert, K. (2014). Automated identification of subcellular organelles by coherent anti-Stokes Raman scattering. Biophys. J. *106*, 1910-1920. https://doi.org/10.1016/j.bpj.2014.03.025.
